# 804. Infection Control and Clinical Support in Long-Term Care Homes During the COVID-19 Pandemic in North Toronto: A Quasi-Experimental Study

**DOI:** 10.1093/ofid/ofab466.1000

**Published:** 2021-12-04

**Authors:** Maria M Magaz, Jaclyn O’Brien, Victoria R Williams, Christina Chan, Adrienne Chan, Natasha Salt, Jerome A Leis

**Affiliations:** Sunnybrook Health Sciences Centre, Toronto, Ontario, Canada

## Abstract

**Background:**

Wave one of the COVID-19 pandemic in Ontario, Canada, resulted in significant institutional outbreaks associated with high case fatality among older adults. Our hospital formally partnered with congregate care homes in north Toronto to support infection control and clinical management before wave two of the COVID-19 pandemic. Our aim was to evaluate the impact of this program on resident and healthcare worker (HCW) outcomes.

**Methods:**

A multicentre quasi-experimental study was conducted comparing outcomes between wave one (March-June, 2020) and wave two (October-December, 2020) among 17 congregate care homes (4 long term care homes and 13 residential homes). During wave two, weekly meetings and 42 on-site visits were conducted along with on-site daily hospital presence for all COVID-19 outbreaks to support infection control and resident management. The primary outcomes included COVID-19-case fatality rate as well as overall resident fatality including COVID-19 and non-COVID-19 related causes. Secondary outcomes included healthcare worker COVID-19 infections, and infection control practices among homes with paired audits (n=6), including hand hygiene, use of personal protective equipment, environmental cleaning and physical distancing practices.

**Results:**

Among 2203 residents during wave one and 2287 residents during wave two, there was reduction in COVID-19 case fatality rate (38.1% vs. 13.4%; p< 0.01), overall COVID-19-related fatality (2.3% vs. 1.0%; p< 0.01) and non COVID-19 related fatality (8.3% vs. 3.5%; p< 0.01). Weekly staff testing and increased syndromic surveillance was implemented during wave two. Among 2590 staff, there were 2.6% vs.4.2% staff who tested positive for COVID-19 during wave one and two, respectively. Changes in infection control practice were observed in regard to directly observed hand hygiene (83.3% vs. 100%), use of personal protective equipment (16.7% vs. 83.3%), environmental cleaning (66.7% vs. 100%) and physical distancing (66.7% vs. 83.3%).

**Conclusion:**

Integration of hospital with community congregate care homes was associated with improvements in resident outcomes during wave two of the pandemic. Further longitudinal support and evaluation is needed to ensure sustainability.

**Disclosures:**

**All Authors**: No reported disclosures

